# Ιnteractions between *Beauveria bassiana* and *Isaria fumosorosea* and Their Hosts *Sitophilus granarius* (L.) and *Sitophilus oryzae* (L.) (Coleoptera: Curculionidae)

**DOI:** 10.3390/insects10100362

**Published:** 2019-10-19

**Authors:** Spiridon Mantzoukas, Athanasia Zikou, Vasw Triantafillou, Ioannis Lagogiannis, Panagiotis A. Eliopoulos

**Affiliations:** 1 Department of Pharmacy, School of Health Sciences, University of Patras, 26504 Patras, Greece; lagoipp@gmail.com; 2 Department of Agricultural Technology, Technological Institute of Western Greece, 27200 Amaliada, Greece; zikoatha69@gmail.com (A.Z.); vaswtriantafyllou@gmail.com (V.T.); 3 ELGO-Demeter, Plant Protection Division of Patras, NEO & L. Amerikis, 26442 Patras, Greece; 4 Department of Agriculture and Agrotechnology, University of Thessaly, 45100 Larissa, Greece; eliopoulos@uth.gr

**Keywords:** interaction, *Beauveria bassiana*, *Isaria fumosorosea*, *Sitophilus granarius*, *Sitophilus oryzae*

## Abstract

The interactions between the entomopathogenic fungus *Beauveria bassiana* Balsamo (Vuillemin) (Hypocreales: Cordycipitaceae) and the entomopathogenic fungus *Isaria fumosorosea* (Wize) Brown and Smith (Hypocreales: Clavicipitaceae) were examined on young adults of *Sitophilus granarius* (L.) (Coleoptera: Curculionidae) and *S. oryzae* (L.) (Coleoptera: Curculionidae). Conidial suspensions of these entomopathogenic fungi were applied both separately and in combination, at three dosages, 10^4^, 10^6^, and 10^8^ conidia/mL. Mortality of experimental adults was recorded daily for 15 days. An overall positive interaction between the pathogenic microorganisms was observed. Mean weevil mortality caused by the separate acting fungi, *B. bassiana,* ranged from 26.7% to 53.3% and from 36.6% to 63.3% for *S. granarius* and *S. oryzae*, respectively. The respective values for *I. fumosorosea* were 20.0%–53.3% and 46.7%–66.7%. The combined treatments showed a distinct interaction between the pathogens; for *S. granarius*, the interaction between the pathogens was additive in all combinations, whereas, for *S. oryzae*, the interaction was additive in seven and competitive in two of the combinations. Applying both entomopathogenic microorganisms may offer a method for weevil control that could be more effective than using each pathogen alone.

## 1. Introduction

*Sitophilus granarius* (L.) (Coleoptera: Curculionidae) and *S. oryzae* (L.) (Coleoptera: Curculionidae) are the most important storage pests of raw cereals throughout the world. To control these pests, synthetic insecticides are used during the storage of grains [1,2]. Considering the dire effects for both humans [3] and the environment [4], health authorities are wary of the use of chemical insecticides on grains [5]. The control of *S. granarius* and *S. oryzae* is currently based mainly on the use of two broad categories of insecticides: residual insecticides and fumigants. Sitophilus species have been reported to develop resistance to synthetic chemicals [6,7]. The development of resistance to these substances and the demands of consumers for residue-free food have led researchers to evaluate the use of alternative control methods that do not leave residues on the product and are generally safe for the environment. 

Insect pathogens, such as the Hypocreales fungi, offer many advantages including high efficacy and compatibility with other IPM methods, and they are thus considered to be among the most promising alternatives to chemical-based insect control [8]. *Beauveria* and *Isaria* are important Hypocreales genera which are being used for insect management. Several valuable findings concerning the use of Hypocreales fungi as an effective control strategy have been documented by various scientists, particularly with special reference to Coleopteran insect pests [9,10]. 

In mixed infections, it is possible that the efficacy of one or both pathogens may be improved, enhanced, or suppressed. It is well established that the fungal efficacy can be enhanced by the simultaneous presence of other insecticidal factors with completely different modes of action [10,11,12,13,14,15,16,17,18,19,20]. Generally, insect infections by more than one pathogen usually lead to an increase in host mortality, particularly when infections are separated by a time interval of several days [15]. 

Additive and synergistic effects of entomopathogenic fungi with other insecticidal treatments have been validated in many previous studies, concerning mainly the combined use with entomopathogenic bacteria [14,18,19,20,21,22], viruses [23], nematodes [16,17,24,25,26], synthetic insecticides [27,28,29,30], and insecticidal dust [10,12]. On the contrary, there are very few studies investigating the combined action of two fungal entomopathogens [31,32,33]. 

It could be assumed that the combination of two similar fungal entomopathogens is unjustified, because they have a common mode of action. However, there are some additional facts that must also be taken into consideration. Firstly, variable insecticidal metabolites and toxins produced by fungal entomopathogens have several modes of action (some of them remain partly unknown) and, in many cases, they often constitute the direct cause of insect death [34,35]. Secondly, similar entomopathogens may act differently on insects with varying behaviors and in different environments [31,32,36,37].

The objective of the present study was to evaluate in vitro interactions between two Hypocreales entomopathogenic fungi when applied against *S. granarius* and *S. oryzae* adults. Such interactions between two fungal infections have not yet been evaluated in terms of pest control efficacy. We aim to investigate whether these two pathogens interact synergistically when applied together at various dose combinations. Our results are discussed on the basis of promoting the use of entomopathogenic fungi as biocontrol agents in storage facilities.

## 2. Materials and Methods 

### 2.1. Insect Rearing

Adults of *S. granarius* and *S. oryzae* that were used in the tests were collected from rearings which had been kept for more than two years in the EMBIA Laboratory of the Pharmacy Department of the University of Patras. Weevils were reared on hard wheat and kept in a growth chamber (PHC Europe/Sanyo/Panasonic Biomedical MLR-352-PE) in controlled environmental conditions (25 ± 1 °C, 65 ± 5% r.h., complete darkness). 

### 2.2. Entomopathogenic Fungi

We used the Hypocreales fungal strains of *Beauveria bassiana* (strain name: *GBBSTTS*) and *Isaria fumosorosea* (strain name: *RHZ4RAS*). These were first isolated from soil samples collected in the prefecture of Achaia using stored pests as baits. The isolates were kept in Petri dishes on the nutrient SDA material (Sabouraud Dextrose Agar, OXOID Ltd., Basingstoke Hampshire, UK) and were renewed every month. The Petri dishes were kept in continuous darkness, at 25 ± 1 °C and 85 ± 5% relative humidity, to enable the incubation of the fungi. The developed fungi were isolated again to avoid infestation and to achieve clear cultivation. 

### 2.3. Conidial Suspensions

Conidia were harvested by scraping the surface of the Petri dishes with a sterilized scalpel and by flooding the dishes with a sterile liquid solution of 0.1% Tween 80 (20 mL per plate). The conidial suspensions were stirred using a magnetic stirrer (Bande Stirrers magnetic stirrer MS300, Bante Instruments Inc., Sugar land, TX, USA) and filtered twice using a sterile cloth. Suspensions were adjusted according to Gurulingappa et al. [38] using a Neubauer hemocytometer (TIEFE 0, 100 mm 1/400 9 mm). Following Goettel and Inglis [39], the viability of conidia was determined after 24 h. The germination test was run for every stock suspension in order to ensure the constancy of the viability assessments. The average viability of conidia was for *I. fumosorosea* 98.7% and *B. bassiana,* 96.9%. Preparation of conidial suspensions and conidial germination took place in a laminar flow chamber (Equip Vertical Air Laminar Flow Cabinet Clean Bench, Mechanical Application Ltd. Athens, Greece).

### 2.4. Bioassays

The virulence of each fungus was investigated separately on *S. granarius* and *S. oryzae* adults which were treated with three different conidial concentrations from *I. fumosorosea* (If) and *B. bassiana* (Bb) (10^4^, 10^6^, and 10^8^ conidia /mL). Each fungus was applied separately. Ten weevil adults (3–5 days old) were collected from lab cultures and transferred in sterile Petri dishes (9-cm diameter) with a single layer of hard wheat grains. Experimental adults were sprayed with 2 mL of the desired conidial suspension using a Potter spray tower (Burkard Manufacturing Co. Ltd., Rickmansworth, Hertfordshire, UK) at 1 kgf cm^−2^. Following this, Petri dishes were kept in incubators set at 25 ± 1 °C and 65% relative humidity during the entire experimental period. Adults were observed daily, and mortality was recorded for 15 days. Adults that were sprayed simply with an aqueous solution with 0.01% Tween^®^ 80 (Sigma-Aldrich^®^, Munich, Germany) were used as control. Each treatment (Petri dish with 10 adults) was replicated 10 times (n = 100 adults for every treatment). The applications of pathogens within each replicate were done at the same time.

The combined effect of the two Hypocreales fungi was tested on adults exposed to all nine different combinations of the three tested conidial concentrations. Experimental adults were initially sprayed with 2 mL conidial suspension of the one pathogen and, after 2 s, they were sprayed with 2 mL conidial suspension of the other pathogen. The spraying sequence (meaning which fungus was applied first) changed from a Petri dish to the next one. This was necessary so as to exclude any variation in our results because of the time of fungus colonization. The experimental procedure (number and age of experimental adults, number of replications, recording of mortality, etc.) were the same as in the case of the separate fungus study (described above). 

### 2.5. Microscopic Fungal Identification Method

Isolates were sub-cultured several times to ensure that purity and monosporic cultures from all isolates were obtained, and they were then morphologically identified by a microscope ZEISS Primo Star (Carl Zeiss Microscopy GmbH, Jena, Germany) at 400× magnification. 

### 2.6. Mathematical Estimation

The interaction between the pathogens was estimated using the formula of Robertson and Preisler [40]:

P_E_ = P_0_ + (1 − P_0_) × (P_1_) + (1 − P_0_) × (1 − P_1_) × (P_2_) (1)

where P_E_ is the expected mortality induced by the combination of the two pathogens; P_0_ is the observed mortality of the control; P_1_ is the observed mortality caused by the first pathogen (separate action); P_2_ is the observed mortality caused by the second pathogen (separate action). Distribution was determined by the chi-square formula:

x^2^ = (L_0_ − L_E_)^2^/L_E_ + (D_0_ − D_E_)^2^/D_E_ (2)

where L_0_ is the number of recorded live larvae of the control, D_0_ is the number of recorded dead larvae of the control, L_E_ is the expected number of live larvae, and D_E_ is the expected number of dead larvae (estimated like P_E_ with Equation (1)). The formula was used to test the hypothesis independent—simultaneous relationship (*df* = 1, *p* = 0.05). If χ^2^ < 3.84, the ratio is defined as additive, if χ^2^ > 3.84 and the observed mortality is higher than expected, the relationship is defined as synergistic. On the contrary, if χ^2^ > 3.84 and the observed mortality is less than expected, the relationship is defined as competitive [14].

### 2.7. Statistical Analysis

Prior to analysis, these mortality values were arcsine transformed. Mortality data were then analyzed by means of univariate ANOVA using the general linear model of the IBM (version 23.0, SPSS Inc., Armonk, NY, USA). In case of significant F values, means were compared using the Bonferroni test. The significance level was set at *p* < 0.05. Kaplan–Meier analysis was also selected to determine the median survival time of *S. granarius* and *S. oryzae* individuals following exposure to the pathogens which had been applied both separately and in combination. Comparison of median survival time was performed using one-way ANOVA (Treatment as Factor) (SPSS v.23.0). 

## 3. Results

### 3.1. Separate and Combined Mortality 

Mean weevil mortality caused by the separate action of *B. bassiana* and *I. fumosorosea* is presented in Table 1. Entomopathogenic fungi induced significantly different levels of mortality on *S. oryzae* compared with *S. granarius* (F: 4.415; df: 1.360; *p*: 0.012) (Table 1). Significant differences in mortality were also detected between the two different pathogens (F: 2.031; df: 5.360; *p*: 0.026). Mortality induced by the entomopathogenic fungi was dose-dependent only in the case of *S. granarius*. More specifically, 15 days after the treatment with *B. bassiana*, the mortality of *S. granarius* adults increased significantly from 27% (10^4^ conidia/mL) to 53% (10^8^ conidia/mL) (F: 4.128; df: 2.90; *p*: 0.008). Similarly, in treatments with *I. fumosorosea*, the weevil mortality ranged from 20% (10^4^ conidia/mL) to 53% (10^8^ conidia/mL) (F: 4.103; df: 2.90; *p*: 0.009) (Table 1). On the contrary, in the case of *S. oryzae*, mortalities did not differ significantly between the various doses of both pathogens (*B. bassiana*: F: 1.665; df: 2.90; *p*: 0.195, *I. fumosorosea*: F: 0.560; df: 2.90; *p*: 0.573) (Table 1). 

As far as the combined action of the two entomopathogens is concerned, a total of nine combined treatments of *B. bassiana* and *I. fumosorosea* were applied against *S. granarius* and *S. oryzae*. Adult mortality of both *S.* *granarius* and *S. oryzae* varied significantly among the various dose combinations (*S. granarius:* F: 1.755; df: 8.270; *p*: 0.086; *S. oryzae*: F: 1.301; df: 8.270; *p*: 0.044) (Table 2). On the contrary, no significant differences in mortality were noted between the two insect species (F: 1.540; df: 1.720; *p*: 0.214) when they were treated with the same combination (Table 2).

The results of the combined treatments showed a distinct interaction between the pathogens. In the case of *S.* *granarius*, the interaction between the pathogens was additive in all combinations (Table 3). On the other hand, pathogens demonstrated an additive interaction when infecting *S. oryzae*, in seven combinations while, in two of the treatments, the interaction was characterized as competitive (Table 3). No synergistic relationship was recorded in any of the combinations.

### 3.2. Fungal Sporulation

**Figure 1:**
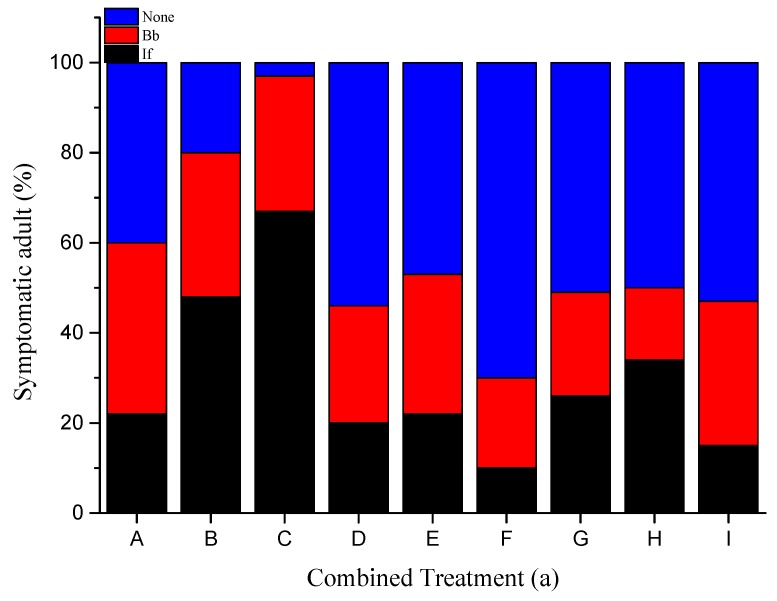

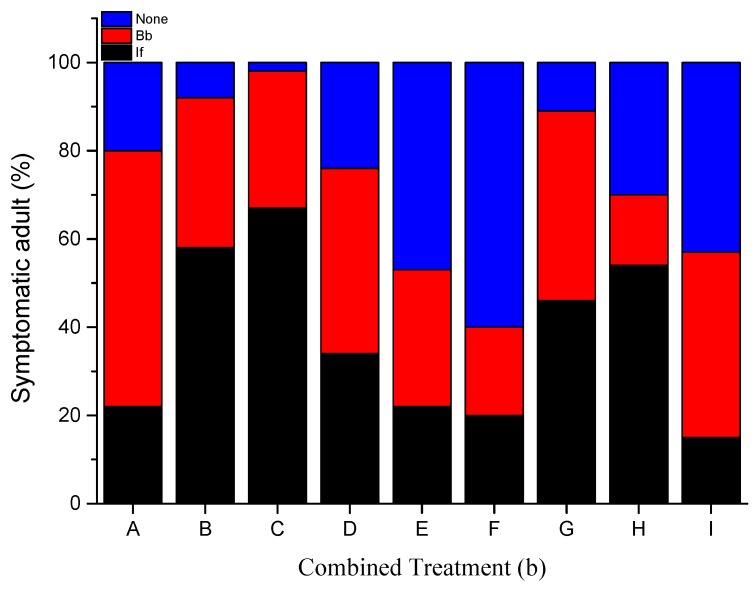
Percent infection attributed to each insect pathogen after the combined inoculation of Hypocreales fungi on the adults of *S. granarius* (**a**) and *S. oryzae* (**b**). If—*I. fumosorosea*; Bb—*B. bassiana*. Combined Treatment: A: 10^8^ Bb × 10^4^ If, B: 10^6^ Bb × 10^6^ If, C: 10^4^ Bb × 10^8^ If, D: 10^8^ Bb × 10^6^ If, E: 10^6^ Bb × 10^8^ If, F: 10^4^ Bb × 10^4^ If, G: 10^8^ Bb × 10^8^ If, H: 10^4^ Bb × 10^6^ If, I: 10^6^ Bb × 10^4^ If.

The percentage of dead insects that did not show sporulation from any of the fungal pathogens varied from 3% to 70% (*S. granarius*) and from 2% to 68% (*S. oryzae*) (Figure 1). The highest fungal sporulation occurred on the cadavers of the combinations B (10^6^ Bb × 10^6^ If) and C (10^4^ Bb × 10^8^ If) in both insects. The two pathogens proved to be equivalent in sporulation, given that cadavers infected with *B. bassiana* were more in half of the combined treatments, with *I. fumosorosea* sporulating more in the other half (Figure 1). The adults that were inoculated with the single treatments of these fungal pathogens exhibited confirmed mortality with the typical fungal symptoms of *I. fumosorosea* and *B. bassiana* in 82% and 79% of the adults, respectively.

### 3.3. Medial Survival Time 

**Figure 2:**
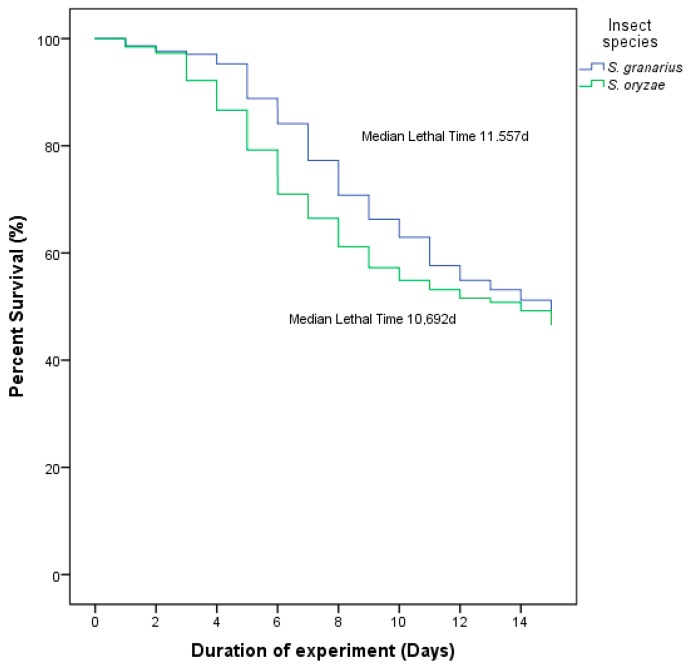
Overall survival of the infected adults of *S. granarius* and *S. oryzae* was monitored for 15 days, after being treated with *B. bassiana* (Bb) and *I. fumosorosea* (If) (chi-square: 7.630; df: 1, *p* = 0.006).

Kaplan–Meier analysis showed that the median overall survival time for experimental adults was 11.557 ± 0.18 days for *S. granarius* and 10.692 ± 0.21 days for *S. oryzae* (Figure 2). The median survival time of *S. granarius* adults with the separate doses was between 9.13 and 12.68 days compared to *S. oryzae* adults whose median lethal time was between 7.97 and 12.65 days (Table 1). For the combined doses of the pathogens, the median survival time of *S. granarius* adults was between 8.00 and 11.8 days compared to *S. oryzae* adults whose median survival time was between 6.36 and 10.37 days in the same combinations (Table 2).

## 4. Discussion

Infective action of entomopathogenic fungi begins when spores are retained on the integument surface and the formation of the germinative tube initiates. Following this, the fungus produces hydrolytic enzymes i.e., proteinases, chitinases, and lipases [41], which enable infection against many Curculionidae [10,42,43,44]. 

Significant differences in mortality caused by the separate action of each entomopathogen were detected not only between the different insects but also between the two fungal entomopathogens. Generally, *I. fumosorosea* was more virulent for *S. oryzae* and *B. bassiana* was more virulent for *S. granarius.* Although mortalities between different host and pathogen species, in our study, did not always differ significantly, it has been well established that these two factors (insect and pathogen species) play an important role in the insecticidal efficacy of entomopathogenic fungi [1,2,32,43,44]. 

An overall positive interaction between the two pathogens was noticed in terms of adult mortality, especially for *S. oryzae*. Based on our results, the interaction of *I. fumosorosea* with *B. bassiana* was additive for *S. granarius* in all combinations. On the contrary, in two treatments, the interaction between the pathogens was negative (competitive) for *S. oryzae*. In our combined treatments, the insect species did not have a significant effect on host mortality. 

Competitive interaction was observed in two of the treatments, in the case of *S. oryzae*. A competitive interaction refers to the negative relationship between pathogens. The nature of competition between entomopathogenic fungi is not well known. The competitive interaction is predominately mediated by resources in the host and the extent to which these benefit the entomopathogens. Mietkiewski and Gorski [45], for instance, report that when the two entomopathogenic fungi are used concurrently with other biological insecticides, they exhibit synergy, competitiveness, or a neutral interaction. Staves and Knell’s [46] findings suggest that if mixed infection persists, then the type of interaction (direct and/or indirect) within the host can play a major role in determining how an entomopathogen will evolve in terms of its virulence. When the combination of two entomopathogenic fungal isolates was tested, all possible effects (additive, synergistic, competitive) were recorded [31,32,33] depending on the host and pathogen species, temperature, and pathogen concentration.

The dead adult percentage showing pink muscardine of *I. fumosorosea* seemed to be higher in 9 of the 18 mixed fungal infections, while the white muscardine of *B. bassiana* on adult cadavers appeared at a higher percentage in the other 9 mixed infections. Our results support Staves and Knell [46] in their suggestion that the dominance outcome during a multiple entomopathogenic fungal infection is not defined solely by the virulence of the entomopathogenic fungi. Moreover, as is reported in an older study, in all the combination treatments with two fungal entomopathogens, only one of the fungi sporulated on the larval cadaver, never both [31].

It is possible to accomplish significant pest control results using a mixture of entomopathogens, either by combining systematically close microorganisms or systematically remote organisms [15]. Several studies have described interactions between different pathogens within the same species [14,18,20,21,22,23,47,48,49,50]. These interactions may affect pest mortality antagonistically (reducing the observed mortality of hosts as compared to single pathogen infections [29]), synergistically (increasing host mortality in comparison to single pathogen infections [16,22,28]), or they may vary per genotype, dose, and order of infection [47,48,51]. Infections of insects by more than one pathogen usually lead to an increase in host mortality [15] as it was proven in most cases in the present study. 

It was our hypothesis that in the interaction between entomopathogenic fungal isolates, one isolate would dominate as more virulent while the less virulent would still play an auxiliary role in the infection process. Insects would thus die from a reinforced infection and the cadavers would exhibit signs of the more virulent fungus. Our results support this theory. 

Our experiment demonstrates that although *B. bassiana* and *I. fumosorosea* are each on their own able to reduce the population of *S. granarius* and *S. oryzae*, the combination of the two could increase host mortality, thus providing a more effective pest control method than using each pathogen alone. The impact of fungal epizootics on host populations can be very dramatic and many attempts have been made to harness this potential for pest-control purposes. 

## 5. Conclusions

We tested the hypothesis that the co-application of *B. bassiana* and *I. fumosorosea* could be used to increase the mortality, which each fungus alone would individually incite in Sitophilus weevils. We observed that the final mortality was greater for the combination treatments than for the separate treatments, in most cases. While this was a relatively simple simulation, it demonstrates the potential value of utilizing fungal “cocktails” as effective biological tools for pest management. As many entomopathogenic fungi will have some impact upon the host’s immune system, understanding the nature of this impact will be critical for understanding the dynamics of the interaction. Nevertheless, further studies are needed to investigate the mechanism of toxicity of such combinations against serious stored pests.

## Figures and Tables

**Table 1:** Mean mortality and median survival time of adults of *S. granarius* and *S. oryzae* treated separately with *B. bassiana* (Bb) and *I. fumosorosea* (If) after 15 days.

Insect	Concentration (conidia/mL)		Mortality		Median Survival Time (Days)			
	Bb	If	(%)	Sd	Estimate	Sd	95% Confidence Interval	
							Lower Bound	Upper Bound
*S. granarius*	0	0	0.00	0.00	15.000a			
	10^4^	0	26.67aA	5.77	12.800bc	0.772	11.286	14.314
	10^6^	0	46.67bA	10.00	11.867b	0.779	10.341	13.393
	10^8^	0	53.33bA	5.77	12.000b	0.721	10.587	13.413
	0	10^4^	20.00aA	0.00	13.700c	0.517	12.687	14.713
	0	10^6^	36.67bA	5.77	12.500bc	0.679	11.168	13.832
	0	10^8^	53.33bA	11.55	10.767d	0.831	9.138	12.395
*S. oryzae*	0	0	3.33	0.00	14.855a	0.145	14.710	15.000
	10^4^	0	36.60aA	11.55	13.567c	0.466	12.654	14.479
	10^6^	0	43.33aA	11.55	12.100b	0.836	10.461	13.739
	10^8^	0	63.33aA	5.77	10.467d	0.895	8.712	12.221
	0	10^4^	46.67aB	5.77	10.433d	1.017	8.439	12.427
	0	10^6^	55.00aB	7.07	10.800d	0.918	9.000	12.600
	0	10^8^	66.67aA	15.28	9.700d	0.880	7.975	11.425

Mean values of different concentration, within the same insect and pathogen, followed by the same small letter are not significantly different; Mean values of different insect, within the same concentration and pathogen, followed by the same capital letter are not significantly different (Bonferroni test, a = 0.05), Estimate values of median time of the same insect followed by the same small letter are not significantly different (Kaplan–Meier, a = 0.05), * Median Survival Time (*S. granarius*: F: 4.667; df: 6; *p* < 0.001, *S. oryzae*: F: 3.589; df: 6; *p* < 0.001).

**Table 2:** Mean mortality and median survival time of adults of *S. granarius* and *S. oryzae* treated with *B. bassiana* (Bb) and *I. fumosorosea* (If) in combination.

Insect	Concentration (conidia/mL)		Mortality		Median Survival Time (Days) *			
	Bb	If	(%)	Sd	Estimate	Sd	95% Confidence Interval	
							Lower Bound	Upper Bound
*S. granarius*	10^8^	10^4^	66.67bcA	11.55	10.100a	0.712	8.704	11.496
	10^6^	10^6^	56.67bA	5.77	11.367b	0.796	9.806	12.927
	10^4^	10^8^	63.33bcA	11.55	11.067b	0.689	9.715	12.418
	10^8^	10^6^	70.00bcA	10.00	9.900a	0.753	8.425	11.375
	10^6^	10^8^	66.67bcA	7.07	9.800a	0.807	8.217	11.383
	10^4^	10^4^	30.00aA	0.00	13.000c	0.609	11.806	14.194
	10^8^	10^8^	86.67cA	7.07	9.300a	0.661	8.005	10.595
	10^4^	10^6^	46.67bA	5.77	11.267b	0.786	9.726	12.807
	10^6^	10^4^	56.67bA	5.77	10.367ab	0.802	9.710	11.939
*S. oryzae*	10^8^	10^4^	46.67abA	5.77	11.767a	0.803	10.192	13.341
	10^6^	10^6^	50.00bA	0.00	11.433a	0.721	10.021	12.846
	10^4^	10^8^	46.67abA	5.77	11.200a	0.918	9.400	13.000
	10^8^	10^6^	80.00cA	5.77	8.300b	0.752	6.827	9.773
	10^6^	10^8^	73.33bcA	5.77	8.500b	0.928	6.682	10.318
	10^4^	10^4^	36.67aA	5.77	12.033a	0.846	10.376	13.691
	10^8^	10^8^	83.33cA	5.77	7.767c	0.718	6.360	9.173
	10^4^	10^6^	56.67bA	11.55	10.187a	0.805	8.610	11.765
	10^6^	10^4^	60.00bcA	10.00	10.133a	0.824	8.518	11.749

Mean values of different combined concentration, within the same insect, followed by the same small letter are not significantly different; Mean values of different insect, within the same combined concentration, followed by the same capital letter are not significantly different (Bonferroni test, *p* = 0.05), Estimate values of median time of the same insect followed by the same small letter are not significantly different (Kaplan–Meier, *p* = 0.05), * Median survival time (*S. granarius*: F: 1.259; df: 8; *p* = 0.032, *S. oryzae*: F: 1.181; df: 8; *p* = 0.021).

**Table 3:** Observed and expected mortality of *S. granarius* and *S. oryzae* adults at the end of the experiment (15 days), treated with both *B. bassiana* (Bb) and *I. fumosorosea* (If) in several combinations, and their interaction (A = Additive, C = Competitive, S = Synergistic) (n = 100).

Combined Concentration (conidia/mL)		Mortality (%) *		χ^2^ (1 df; *p* = 0.05)	Interaction
Bb	If	Observed	Expected		
*S. granarius*					
10^8^	10^4^	67	66	−0.012	**A**
10^6^	10^6^	57	66	0.138	**A**
10^4^	10^8^	63	63	0.034	**A**
10^8^	10^6^	67-	80	0.520	**A**
10^6^	10^8^	70	70	0.008	**A**
10^4^	10^4^	30	41	−0.161	**A**
10^8^	10^8^	80	78	−0.280	**A**
10^4^	10^6^	46	54	0.019	**A**
10^6^	10^4^	57	57	0.004	**A**
*S. oryzae*					
10^8^	10^4^	77	81	0.366	**A**
10^6^	10^6^	51	75	7.917	**C**
10^4^	10^8^	78	80	0.070	**A**
10^8^	10^6^	73	83	1.986	**A**
10^6^	10^8^	67	84	2.607	**A**
10^4^	10^4^	37	62	8.172	**C**
10^8^	10^8^	89	83	0.984	**A**
10^4^	10^6^	60	72	2.375	**A**
10^6^	10^4^	57	66	1.164	**A**

* Expected mortality is calculated according to Robertson and Preisler [40].
